# *Strychnos nux-vomica* L. seed preparation promotes functional recovery and attenuates oxidative stress in a mouse model of sciatic nerve crush injury

**DOI:** 10.1186/s12906-020-02950-3

**Published:** 2020-06-11

**Authors:** Aroona Razzaq, Ghulam Hussain, Azhar Rasul, Jiaqi Xu, Qiqi Zhang, Shoaib Ahmad Malik, Haseeb Anwar, Nimra Aziz, Nady Braidy, Jose-Luis Gonzalez de Aguilar, Wei Wei, Jiang Li, Xiaomeng Li

**Affiliations:** 1 The Key Laboratory of Molecular Epigenetics of MOE, Institute of Genetics and Cytology, Northeast Normal University, Changchun, China; 2 Department of Physiology, Faculty of Life Sciences, Neurochemicalbiology and Genetics Laboratory (NGL), Government College University, Faisalabad, Pakistan; 3 Department of Zoology, Faculty of Life Sciences, Government College University, Faisalabad, Pakistan; 4 Stomatological Hospital,, Guangzhou Medical University, Guangzhou, China; 5 Centre for Healthy Brain Ageing, School of Psychiatry, The University of New South Wales Sydney, Sydney, Australia; 6 INSERM, U1118, Mécanismes Centraux et Péripheriques de la Neurodégénérescence, Université de Strasbourg, UMR_S 1118, 68000 Strasbourg, France; 7 Department of Biochemistry, Sargodha Medical College, University of Sargodha, Sargodha, Pakistan

**Keywords:** Peripheral nerve injury, *Strychnos nux-vomica*, Functional recovery, Muscle atrophy, Oxidative stress, Glucose tolerance

## Abstract

**Background:**

Peripheral nerve injury is a debilitating condition that may lead to partial or complete motor, sensory and autonomic function loss and lacks effective therapy until date. Therefore, it is quite imperative to explore impending remedies for rapid and accurate functional retrieval following such conditions. Natural product-based intervention can prove effective to facilitate the process of functions regain.

**Methods:**

Here, we investigated the effect of processed *Strychnos nux-vomica* seeds at a dose of 250 mg/kg body weight in a mouse model of induced Sciatic nerve lesion in promoting the recovery of the functions. A compression injury was induced in the Sciatic nerve of the right leg in the mice. Sensory function recovery was evaluated by hot-plate and formalin tests, whereas the motor function retrieval was assessed by measuring muscle grip strength, sciatic functional index, and muscle mass restoration. Oxidative stress and blood cell count were measured by biochemistry and haematological analyses.

**Results:**

This study indicates that *Strychnos nux-vomica* seeds enhance the rate of recovery of both sensory and motor functions. It helps restore the muscle mass, attenuates total oxidant status and enhances the total anti-oxidant capacity of the biological system. Moreover, the treated animals manifested an enhanced glucose tolerance aptitude and augmented granulocyte and platelet counts. Improved oxidant control, enhanced glucose sensitivity and amended granulocyte and platelet counts are likely to contribute to the advantageous effects of *Strychnos nux-vomica*, and warrant further in-depth studies for deciphering possible mechanisms and identification of active constituent(s) responsible for these effects.

**Conclusion:**

*Strychnos nux-vomica* seed offers functional recovery promoting effects following a mechanical injury to the Sciatic nerve and the possible reasons behind this effect can be reduced oxidative stress and improved glycaemic control. Further and detailed investigations can unravel this mystery.

## Background

Peripheral nerve injury (PNI) is among the most common health issues of our society and remains incurable to date. It results from several causes such as road accidents, gunshot wounds, sharp lacerations, and other physical traumas. The affected nerve undergoes an intricate series of pathological modifications and often results in a partial and complete loss of both sensory and motor functions. Although the peripheral nervous system (PNS) possesses an ability of self-repairing after an injury, this process of regeneration is terribly sluggish that subsequently exacerbates the muscular atrophy before re-innervation of the denervated muscles occurs [1, 2]. Despite a remarkable work on this aspect, a complete and accurate functional recovery yet remains a challenge. Among other multifarious factors, the occurrence of muscular atrophy and lethargic regeneration rate appear major obstacles in attaining a complete functional recovery [3]. Therefore, there is a growing need for identifying and develop novel therapeutic strategies to stimulate this regeneration process. Plants and plant-derived compounds are attaining much attention due to their unbeatable advantages such as abundant availability, affordability, and negligible side-effects [4–6]. Therefore, scientists are evaluating such natural compounds that can speed-up the process of function retrieval and promote axonal regeneration [7]. Hence, naturally occurring molecules present a promising alternative therapeutic strategy.

*Strychnos nux-vomica* L. (*N. vomica*) is a medium-sized deciduous tree (Family: *Loganiaceae,* genus: *Strychnos*) which is commonly known as Kuchla in the subcontinent region. It is native to South-East Asia and is also cultivated in various other countries of the world including Pakistan. It is a famous nerve stimulant and also exhibits a variety of other pharmacological properties such as anti-oxidant, anti-inflammatory, and anti-nociceptive [8]. Traditionally, it has been an auspicious drug for the treatment of various diseases such as bronchitis, gonorrhoea, and diabetes in Ayurvedic and Unani systems of medicines. The two most important alkaloids found in *N. vomica*; brucine and strychnine are known as potent stimulators of the spinal cord [9]. The hydro-methanol extract of *N. vomica* leaves showed analgesic activity in rats at different doses of 100, 200 and 400 mg/kg [10, 11]. Brucine directly targets the sodium channels and causes their inhibition and therefore is involved in both acute and chronic pain attenuation in a rodent model [12]. Moreover, it enhances acetylcholine binding to the muscarinic 1 receptor, making it a good candidate for the development of drugs against neurodegenerative disorders such as Parkinson’s disease (PD) and Alzheimer’s diseases (AD) [13]. The potential therapeutic properties of *N. vomica* against PD, muscular rigidity, trigeminal neuralgia and epilepsy have been reported [14]. Similarly, its antiepileptic effects in a study conducted on mice have also been investigated [15].

It has been used as a nerve tonic to improve a slow pulse rate. Its methanolic extract is effective in increasing the activity of antioxidant enzymes such as superoxide dismutase (SOD) and catalase in diabetic rats. It suggests that the anti-oxidative activity of *N. vomica* might interfere with the elevated oxidative stress (at the nerve injury site) to facilitate nerve regeneration [16, 17]. Based on the available data, one can speculate that *N. vomica* might play a protective role for the injured fibres of PNS, as previously reported for the CNS [18].

Taking into account the reported shreds of evidence regarding the effects *N. vomica* on the nervous system, we were curious in investigating its effects on the PNS. In such circumstances, the animal model appears the most acceptable and valuable tool to conduct such kind of study. Since the injury to a peripheral nerve is the most commonly observed condition, therefore, we took a privilege of our already established mouse of Sciatic nerve lesion to explore the possible role of *N. vomica* in the case of PNI.

## Methods

### Animals

Adult male albino mice (*n* = 14) having similar weight (30 ± 4 g) and age (6–7 weeks) were used in this study. They were procured from the Department of Physiology, Government College University Faisalabad, Pakistan. The mice were divided into two groups (Normal chow group *n* = 7 and *N. vomica* chow group n = 7) and were housed in standard management conditions (one mouse/cage, 12-h dark/12-h light cycle, 24 ± 2 °C) with ad libitum food and water supply. At the end of the experiment, the mice of both groups were euthanized by decapitation after giving deep anesthesia by injecting ketamine (70 mg/Kg body weight) and xylazine (5 mg/kg body weight), blood was collected and tissue samples were harvested. The study was approved by the Institutional Animal Care and Ethics Committee.

### Sciatic nerve compression injury

Sciatic nerve compression injury was performed as described previously [19]. Briefly, the mice of both groups were anesthetized by intraperitoneal injection of ketamine (70 mg/Kg body weight) and xylazine (5 mg/kg body weight). To induce injury, the sciatic nerve at mid-thigh region was exposed and the injury was induced before the nerve bifurcates. This process was performed on the right hind limb (Ipsilateral) while the left one was left unoperated (Contralateral). Following the induction of injury in all mice, they were divided into two groups; normal chow group (*n* = 7) and *N. vomica* chow group (n = 7).

### Plant material preparation and dosage

The plant was purchased from the local market and its identification was confirmed by the Department of Botany, Government College University Faisalabad (Herbarium Number 246-bot-2019). The detoxification and preparation of plant material were adopted with some modifications from the procedure described by M. Kabir-u-din (Mokhazan al mafardat), Bhati et al.*,* Anwar et al.*, and* Kashani et al [11–13, 20]*.* In brief, the seeds were purified by soaking in the water for 15 days and the water was changed daily. Then seeds were peeled off, the central toxic part of the seeds (embryo) was removed and the remaining parts of seeds (dicotyledon) were shade dried. The dried seeds were ground into a fine powder of 60 mesh size and mixed in the rodent chow diet at a dose of 250 mg/kg body weight throughout the study. The dose adjustment was done according to the literature providing shreds of evidence for *N. vomica* after detailed research from web sources [1–3]. Briefly, it was ensured that an average daily dietary intake of 5 g comprised of the required dose of plant material. The dose comprising diet was offered to the mice of *N. vomica* chow group from the day of injury during the whole period of study.

### Muscle grip strength

It is a non-intrusive, in vivo method, used to measure the muscle strength by placing the mouse on a metal bar or horizontal grid (Bioseb, Chaville, France). Muscle strength was measured for both hind limbs; ipsilateral and contralateral. A mean of 3 readings with a 30 s latency period was taken for each mouse and the data of the treatment group were compared to the normal group to evaluate the functional recovery [14, 19]. A time interval of five minutes was taken between successive readings for the same animal.

### Sciatic functional index

The Sciatic Functional Index (SFI) was evaluated using the method described by Ma et al., 2016 [21]. Briefly, the hind paws were painted with blue ink and the mouse was allowed to walk on a 50 cm long wooden track with a white paper on its floor. The clearest prints per run were selected for the measurement of SFI as normal (N) and experimental (E) paw. The following formula was used to calculate the SFI:

$$ SFI=\left(-38.3\times -\frac{\mathrm{EPL}-\mathrm{NPL}}{\mathrm{NPL}}\right)+\left(109.5\times \frac{\mathrm{ETS}-\mathrm{NTS}}{\mathrm{NTS}}\right)+\left(13.3\times \frac{\mathrm{EIT}-\mathrm{NIT}}{\mathrm{NIT}}\right)-8.8 $$

The distance between the heel and top of the third toe is the print length (PL), the distance between fifth and first toe is toe spread (TS), and the distance between fourth and second toe is intermediary toe spread (IT). EPL, ETS, and EIT are the PL, TS and IT in ipsilateral (experimental) paw and NPL, NTS, NIT are PL, TS and IT in contralateral (normal) paw.

### Hot-plate test

Sensory function retrieval was evaluated by performing a hot-plate test (SCILOGEX MS7-H550-S LED digital 7 × 7 Hotplate stirrers). It was performed in a similar manner as previously reported [22]. The mice were adapted to the non-functioning device for 1 min prior to actual testing. Each mouse was placed to stand in a position so that its operated paw was exposed to the hot surface of the plate set at a temperature (56 + 2 °C) until the mouse licked or jumped its paw. This value was taken as hot-plate latency (HPL) and the mouse was instantly removed following any reaction. The withdrawal reflex was noted and 3 readings were recorded with 2-min intervals between successive readings. If there was no response for 30 s, the thermal stimulus was stopped to avert tissue injury and withdrawal reflex was measured as 30 s.

### Paw withdrawal threshold

The paw withdrawal threshold in response to a chemical stimulus was measured two times; pre and post-injury. Formalin (10 μl of 5%) was injected on the dorsal surface of the operated hind paw with 50 μl Hamilton syringe [23]. The mice were then placed in a plastic cage and were observed for 5 min to record their activity of first lick or jerk.

### Total oxidant status (TOS)

The TOS level was measured using the Erel TOS method [24], based on the oxidation of the ferrous ion into ferric ions in the presence of oxidant species under acidic environment. The ferric ion conversion was measured by using xylenol orange with a semi-automated chemistry analyser (Biosystem, BTS-330). The test parameters were as follows: temperature, 37 °C; primary wavelength, 560 nm; secondary wavelength, 800 nm; reagent 1 volume, 225 μL; serum sample, 35 μl; reagent 2 volume, 11 μL; and reaction time, 4 min. The results were expressed in μmol H_2_O_2_ equivalent/L (μmolH_2_O_2_ Equiv/L).

### Total antioxidant capacity (TAC)

The TAC was measured using the most common direct assay method; Trolox Equivalent Antioxidant Capacity (TEAC). It is based on the 2,2′-azino-di-3-ethylbenzthiazoline sulfonate (ABTS+) colorimetric method. This assay directly depends on the capability of antioxidants in serum sample to inhibit the formation of ABTS• + following the incubation of ABTS with H_2_O_2_. The 5 mM Trolox solution was used as a standard for the calculation of antioxidants in the serum sample using a semi-automated chemistry analyser (Biosystem, BTS-330). The test parameters were as follows: temperature, 37 °C; reagent 1 volume, 200 μL; serum sample, 5 μL; reagent 2 volume, 20 μL; reaction time, 5 min; and monochromatic wavelength, 660 nm. The results were expressed in mmol Trolox equivalent/L (mmol Trolox equiv./L) [25].

### Paraoxonase activity (PON-1; U min-1 ml-1)

Paraoxonase 1 (PON-1) is a hydrolytic enzyme with an intrinsic capability to protect the living system against oxidative stress-induced damages. PON-1 is an antioxidant factor and its activity depends on the degree of enzymatic degradation of paraoxon into the p-nitrophenol in the presence of H_2_O. The activity of PON-1 was determined by measuring an increase in the absorbance of p-nitrophenol on a semi-automated chemistry analyser (Biosystem, BTS-330). The mixture of 0.10 M Tris-HCL buffer (8.0 pH) having calcium chloride (2 mM) with the paraoxon (2 mM) was used. The paraoxon was taken as a substrate, used to evaluate the enzymatic activity of PON1. The test parameters were as follows: temperature, 37 °C; reagent volume, 1050 μL; serum sample, 30 μL; wavelength, 405 nm; and reaction time, 1 min. The first reading was taken just after adding the sample with reagent and the second reading was taken after the incubation of 1 min. The delta change in the absorption was recorded and the results were expressed as PON-1; U min-1 ml-1 [26, 27].

### Arylesterase activity (ARE; KU min-1 L-1)

The activity of Arylesterase (ARE) is associated with the antioxidant capacity of the biological system. In this assay, phenylacetate was used as a substrate that is hydrolysed into phenols in the presence of ARE. Phenylacetate was mixed with methanol (40%) for making the stock solution and the mixture comprised of 0.1 M Tris-HCL Buffer (8.0 pH) having calcium chloride (2.0 mM) and phenylacetate (2.0 mM). The activity of ARE was determined by measuring an increase in the hydrolysis rate of phenols on a semi-automated chemistry analyser (Biosystem, BTS-330). The test parameters were as follows: temperature, 37; reagent volume, 350 μL; serum sample, 10 μL; wavelength, 270 nm; and reaction time, 1 min. The first reading was taken just after adding the sample with reagent and the second reading was taken after the incubation of 1 min. The delta change in absorption was recorded and the results were expressed as ARE; KU min-1 L-1 [28].

### Muscle mass

The extent of muscle fibres atrophy was measured by comparing the muscle mass. The Gastrocnemius and Tibialis anterior muscles from both normal chow group and *N. vomica* chow group were used for muscle mass assessment [29–31]. The muscle mass ratio of both groups (ipsilateral to contralateral) was compared at the termination of the experiment to detect the substantial difference between treated and untreated mice.

### Random blood glucose

A high level of blood glucose is associated with the development of metabolism-related pathological changes that aggravates the situation during an injury to a nerve. The glycaemic level was measured with a glucometer (Accu-chek). For this purpose, the blood sample was taken from the tail of mice and glucose level was measured by using the procedure described in earlier studies [32–34].

### Glucose tolerance test (GTT)

Glycaemic control was examined using the Glucose Tolerance Test (GTT). The mice were kept on overnight fasting (approximately 12 h) before the beginning of the experiment. The intra-peritoneal glucose shoot (1 g/Kg of body weight) was injected to all the animals in both groups. The blood sample was taken from the tail of mice, and blood glucose was measured at time point 0 min (before glucose shoot) and subsequent measurements were taken at 15, 30, 60, 90 and 120 min [35]. The GTT was done on two separate occasions after 1 week and 2 weeks of *N. vomica* chow diet provision.

### Haematology analysis

Following the decapitation, blood sample was collected and blood cell count was measured using haematology analyser (Norma ICON 3). Platelets and various types of white blood cells were measured because of their copious roles in promoting the axonal regeneration in the nervous system [36, 37].

### Statistical analysis

All data are represented as mean ± standard error of the mean (SEM). Statistical analysis was performed with Prism 7.0a (GraphPad, San Diego, CA). Unpaired t-test was used to compare the means of two experimental conditions. To compare the means of more than two experimental conditions, we used 2-way repeated-measure ANOVA followed by pairwise comparisons with Benjamini-Hochberg correction. Differences with *P*-values of less than 0.05 were considered significant.

## Results

### Effects of *N. vomica* on food consumption and body mass

**Fig. 1:**
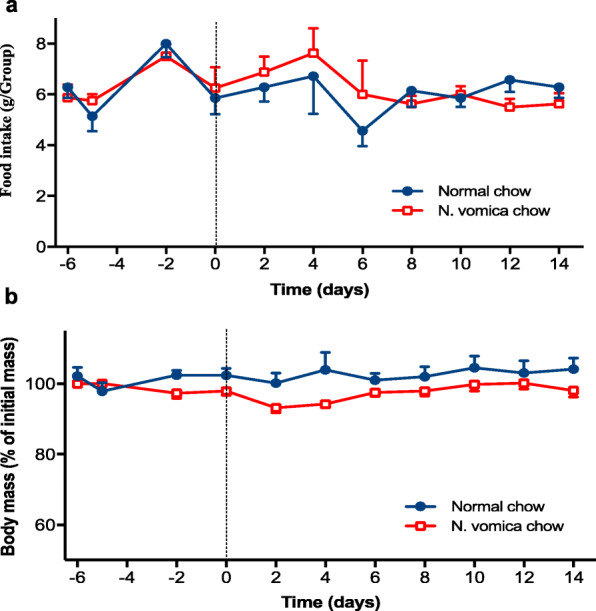
*N. vomica* supplementation does not alter food intake and body mass. **a** Time course of food intake in the mice fed on normal chow (blue circles, n=7) or *N. vomica* chow (red squares, n=7). The mice started taking *N. vomica* chow at the time of sciatic nerve crush (dotted line at day 0) and during the whole period of study. Two-way repeated-measure ANOVA (diet x time) showed a significant effect of time (F_(10,130)_=2.44, *P*=0.01), a non-significant effect of diet (F_(1,13)_=0.05, *P*=*0.824*) and a non-significant interaction between factors (F_(10,130)_=0.7, *P*=*0.72*). **b** Time course of body mass of the mice as in **a**. Body mass is expressed as a percentage of initial mass at days -6 and -5 (before injury) per individual. Two-way repeated-measure ANOVA (diet x time) showed non-significant effects of diet (F_(1,13)_=3.84, *P*=0.071) and time (F_(10,130)_=1.72, *P*=*0.081*), and a non-significant interaction between factors (F_(10,130)_=1.68, *P=0.09*)

To evaluate the effect of *N. vomica* on food intake and body mass, we measured both parameters throughout the experiment (before and after nerve injury) in both group’s i.e. normal chow and *N. vomica* chow group (Fig. 1a). The food intake was measured daily at a fixed time interval by subtracting the present quantity of the food from the food quantity of yesterday. We found that the presence of *N. vomica* in the diet did not hamper the mice’s eating behaviour, suggesting that its addition did not change the smell, granularity, and taste of the food *(P < 0.72)*. The body mass slightly decreased (Fig. 1b) in the *N. vomica* chow group but this reduction did not touch a statistical significance level *(P < 0.09)*. Additionally, neither the body mass nor the food intake was modified before and after the surgical procedure, which further validates the responsiveness of the mice to the treatment. Based on these observations, it can be speculated that the obtained results were exclusively attributed to *N. vomica*.

### *N. vomica* accelerates motor function recovery

**Fig. 2:**
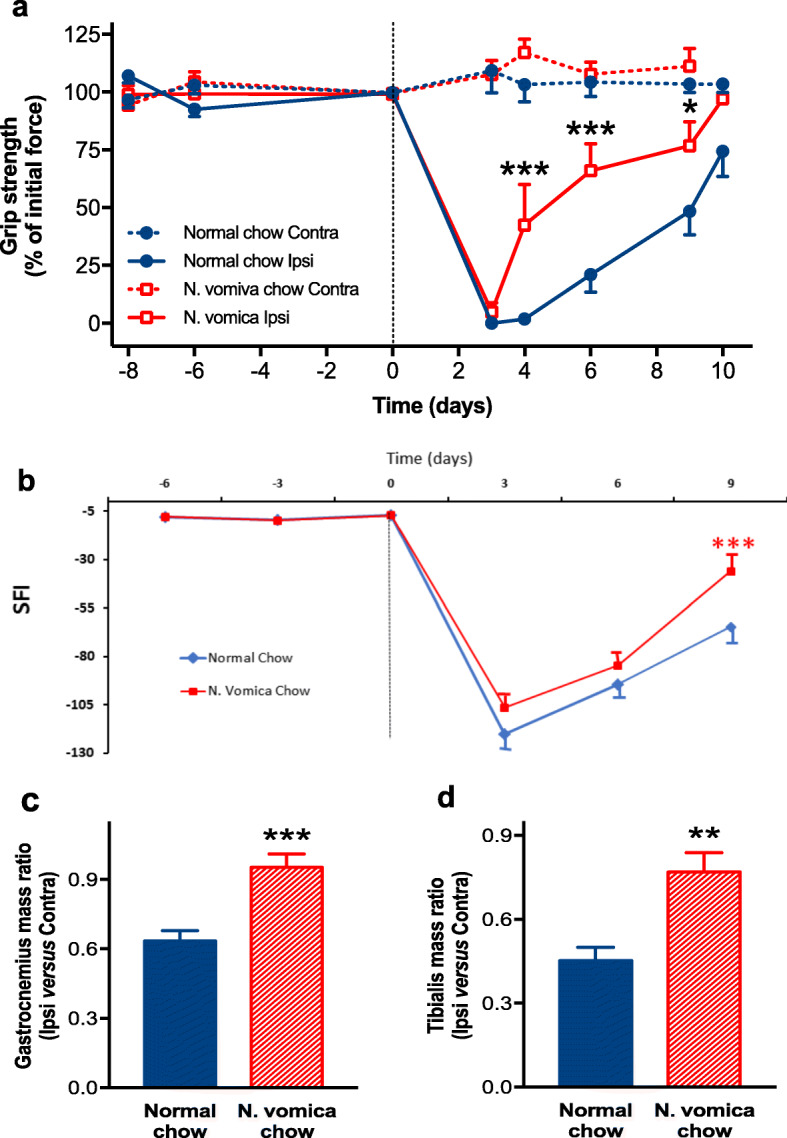
*N. vomica* accelerates motor function recovery after nerve injury. **a** Time course for muscle grip strength in the mice fed on normal chow (blue circles, n=7) or *N. vomica* chow (red squares, n=7). The mice were fed on *N. vomica* chow diet at the time of sciatic nerve crush (dotted line at day 0) and during the whole period of function recovery. Measurements were obtained from both contralateral (dotted lines) and ipsilateral (solid lines). Grip strength is expressed as a percentage of initial force developed at days -8 and -6 (before injury) per individual. Two-way repeated-measure ANOVA (diet x time) showed significant effects of diet (F_(1,13)_=6.63, *P*=0.023) and time (F_(7,91)_=63.42, *P*<0.0001), and a significant interaction between factors (F_(7,91)_=4.17, *P*=0.0005). Post-hoc pairwise comparisons with Benjamini-Hochberg correction revealed significant differences between normal and *N. vomica* at 4, 6 and 9 days after lesion (*P<0.05; ***P<0.001). **b** Time course of the sciatic functional index (SFI) of mice as in a. Two-way repeated-measure ANOVA (diet x time) showed a significant effect of time. Post-hoc pairwise comparisons with Benjamini-Hochberg correction revealed significant differences between normal and *N. vomica* chow at 9 days after lesion (***P<*0.001*). Gastrocnemius (**c**) and Tibialis (**d**) muscle mass in mice as in **A**. Measurements were obtained after sciatic nerve crush and subsequent functional recovery, and they are expressed as a ratio between ipsilateral and contralateral. Unpaired t-test showed a significant effect of diet on both muscles (**P<*0.01*; ***P<*0.001*)

The pattern of motor function retrieval after sciatic nerve crush in mice was evaluated by grip strength test (% of initial force) and SFI. *N. vomica* promoted retrieval of motor function both in the term of muscle grip strength (***P < *0.001*) and walking pattern (***P < *0.001*) (Fig. 2a and b). The target muscle undergoes atrophy of fibers as a consequence of interrupted electrical signals. A decrease in muscle mass is an indication of atrophy in response to denervation and vice versa [30, 38]. The mass of Gastrocnemius and Tibialis anterior muscles of the ipsilateral limb was restored in the *N. vomica* chow group (***P < *0.001* and **P < *0.01*) respectively (Fig. 2c and d), whereas muscle atrophy was still noticeable in both muscles of the normal chow group mice. Taken together, these observations put credibility to *N. vomica* that it efficiently re-establishes muscle mass to the normal level.

### *N. vomica* accelerates sensory function recovery

**Fig. 3:**
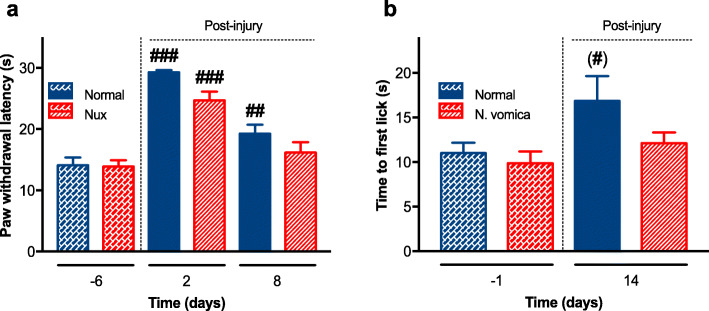
*N. vomica* promotes nociceptive sensation after nerve injury. **a** Paw withdrawal latency in response to thermal stimulus in normal chow (blue columns, n=7) or *N. vomica* chow (red columns, n=7) groups. Measurements were obtained before and after the crush injury. Two-way repeated-measure ANOVA (diet x time) showed a significant effect of time (F_(2,26)_=61.83, *P*<0.0001), a non-significant effect of diet (F_(1,13)_=4.61, *P*=0.051) and a non-significant interaction between factors (F_(2,26)_=1.69, *P*=0.203). Post-hoc pairwise comparisons with Benjamini-Hochberg correction revealed significant differences at day 2 and 8 after lesion in normal chow-fed mice and at day 2 after lesion in *N. vomica* chow-fed mice (^##^*P<0.01*; ^###^*P*<*0.001*). **b** Time to first lick in response to formalin application as in **a**. Two-way repeated-measure ANOVA (diet x time) showed a significant effect of time (F_(1,13)_=6.01, *P*=0.029), a non-significant effect of diet (F_(1,13)_=2.89, *P*=0.112) and a non-significant interaction between factors (F_(1,13)_=1.19, *P*=0.295). Post-hoc pairwise comparisons with Benjamini-Hochberg correction revealed significant differences at 14 days after lesion in normal chow-fed mice (^#^*P<0.05*; brackets indicate that the correction *Q*-value, equal to 0.061, almost attained significance)

The regain of sensory function was measured by performing the hot-plate and the formalin tests (Fig. 3a and b). The paw withdrawal latency in the hot-plate test and time to first lick in the formalin test is indicative of the nociception response [39]. The results show that *N. vomica* accelerated sensory function retrieval (^*###*^*P < 0.001,*^*#*^*P < 0.05*) respectively. The reduced paw withdrawal latency and time to the first lick in the treatment group clearly indicate the effect of *N. vomica* on the restoration of nociceptive activity following a lesion.

### Effects of *N. vomica* on oxidative stress and glucose tolerance

**Fig. 4:**
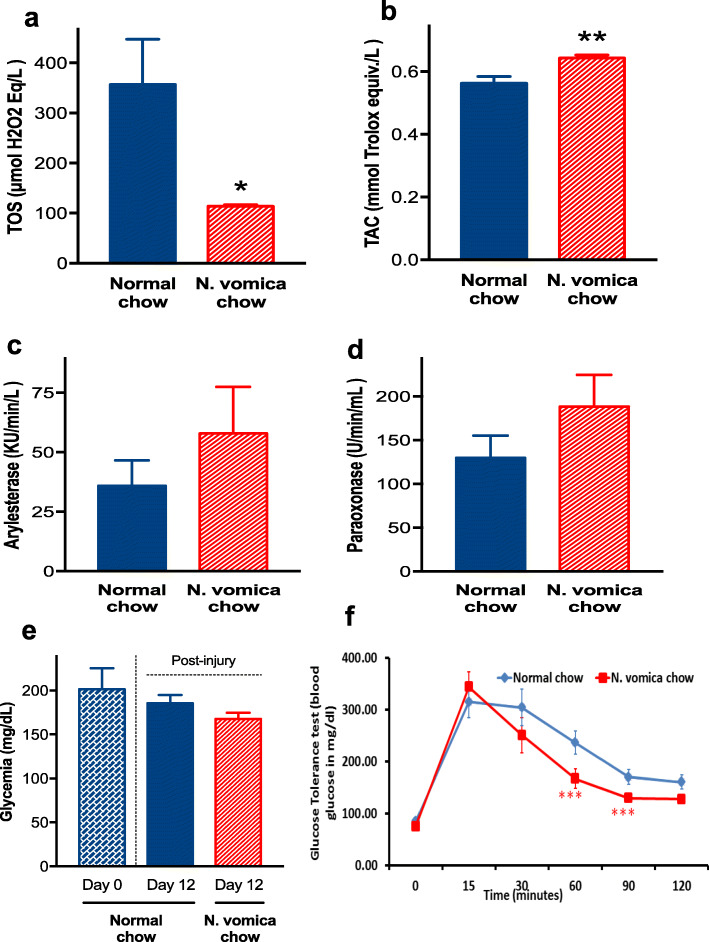
*N. vomica* reduces blood glucose and oxidative stress. An unpaired t-test on TOS (**a**) and TAC (**b**) parameters showed a significant effect of *N. vomica* on oxidative stress reduction (*P<0.05; **P<0.01). Enzymatic activity of arylesterase (**c**) and paraoxonase (**d**) was non-significant. **e** Glycaemic levels at the time of nerve crush (day 0, blue hatched column, n=7) and after 12 days in normal chow (blue solid column, n=7) or *N. vomica* chow (red hatched column, n=7) groups. An unpaired t-test on post-injury measurements did not show any significant effect. **f** Glucose Tolerance Test at week 2 of *N. vomica* supplementation (red line) showed enhanced glucose sensitivity at 60 and 90 minutes of post glucose administration (***P<0.04)

The level of oxidative stress (TAC and TOC) and its related enzyme activity was measured to find out the effect of treatment. *N. vomica* significantly attenuated the level of TOS (Fig. 4a) and enhanced the TAC of treated animals (Fig. 4 b). Similar trends were observed for oxidative stress-related enzymes i.e., *N. vomica* chow group showed the higher activity of ARE (Fig. 4c) and PON-1 (Fig. 4d), although it was statistically non-significant. The balance between oxidative and anti-oxidative processes is sensitive to glucose level as hyperglycemia-induced oxidative stress is a well-recognized entity as a driving force for the development of numerous pathologies [40]. In this context, we measured random glucose levels and found that the *N. vomica* diet group displayed a better glucose handling capacity but the difference was statistically non-significant (Fig. 4e). Further, we assessed the metabolic glycemic control by intra-peritoneal GTT and found that glucose levels significantly decreased in the treatment group (***P < 0.04) as compared to the control group. The *N. vomica* chow group demonstrated a significant glucose clearance in 2nd week as shown in Fig. 4f, while after 1 week of supplementation the results were not significant (data not are shown).

### Effects of *N. vomica* on blood physiology

**Fig. 5:**
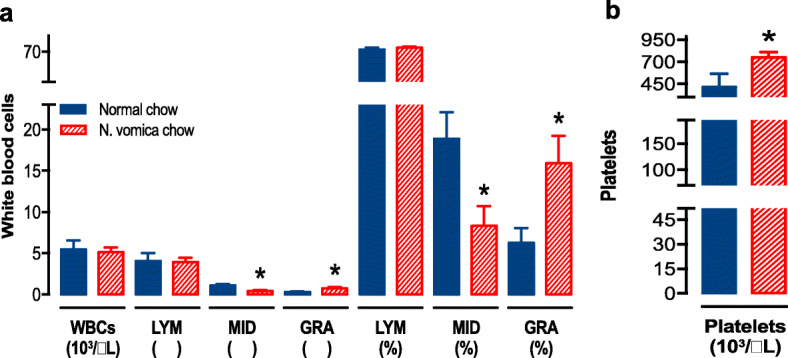
Effect of *N. vomica* on the blood cell count. White blood cell (**a**) and platelet count (**b**) in the mice fed on normal chow (blue solid columns, *n* = 7) or *N. vomica* chow (red hatched columns, n = 7). An unpaired t-test on each parameter showed a significant effect of *N. vomica* (*P < *0.05*). WBC: White Blood cells, LYM: Lymphocytes, GRA: Granulocytes.

Here, we evaluated the effect of *N. vomica* on the various types of blood cells. We found that *N. vomica* raised the number of granulocytes amongst white blood cell populations (Fig. 5a) whereas circulating MID cells, which includes monocytes, were reduced in the animals fed on *N. vomica* (Fig. 5a). Interestingly, platelet count (Fig. 5b) also increased statistically in the *N. vomica* chow group (*P < 0.05).

## Discussion

Peripheral nerve injury (PNI) is a condition with an extensive range of symptoms depending on the nature and severity of injury to the nerve. It affects a huge number of population worldwide and is among the most complex medical conditions to be treated. PNI can consequence both sensory and motor functions disruption, leading to social stigmatization and even life-long dependency. Although, the peripheral nerves manifest an aptitude to regrow and reinnervate the affected muscle after an injury, but complete functions regain may take from several weeks to years depending on the degree of injury [3]. This terrifically slow process of nerve regeneration most often results in the distal muscles’ atrophy and ultimately aggravates the condition. This sluggish rate of regeneration is supposed to be a major player underlying the control of complete and accurate functional regain under ordinary situations. Thus, the escalation of the rate of regeneration of an injured nerve is a dire need of the time for a complete and perfect functional rescue to curtail the muscular atrophy aggravation. Despite the extensive work on this aspect, an affordable and reliable treatment that can ensure a complete function recovery is scarce yet. Since the beginning of human history, plant-based remedies have been practiced in disease curing and they still appear as the most appropriate choice even in this modern era. We have already reported some plants that promote the earlier functional reclamation following a nerve injury. Even if these studies are preliminary steps but, they account for the ultimate hope for accelerating the regeneration and functional rehabilitation [1, 2, 7]. The present work is an extension of our preliminary and ongoing efforts to explore the possible role of natural compounds to improve the functional reclamation following PNI. Here, we investigate the role of a widely used medicinal plant; *Strychnos nux-vomica L.* [6] on the recovery of the function in a rodent model following an induced mechanical insult to the Sciatic nerve.

*N. vomica* has been used as an auspicious drug for the treatment of various diseases such as bronchitis, gonorrhoea, and diabetes [10] in Ayurvedic and Unani systems of medicines. It is a famous nerve stimulant and also exhibits a variety of other pharmacological properties such as anti-oxidant, anti-inflammatory, and anti-nociceptive [8]. Moreover, *N. vomica* has also been investigated for its effectiveness against AD and PD, and as a neuroprotective agent [13]. We found an earlier reclamation of both motor and sensory functions in *N. vomica* treated animals. We did not observe any effect of *N. vomica* addition on both food intake (*P < 0.072*) and body mass (*P < 0.09*) of treated animals. From the observation, we could speculate that an accelerated function recovery was merely due to *N. vomica’s* presence in the diet. The motor function retrieval is connected with the rate of nerve regeneration as the improved number of motor units increases the target muscles’ innervation and hereby causes an effect on the muscle grip strength and SFI. The improvement in muscle grip strength was observable even on day 4 (P < 0.001***). The effect of *N. vomica* on SFI became noticeable on day 3 and this difference appeared significant on day 9 (P < 0.001***). A poor transfer or absence of the basal stimulus to the target muscle, after abrupt injury, leads to denervation-associated muscle atrophy. This causes an additional burden on the nerve injury site and results in depressed functional recovery [38]. At the same time, muscular atrophy, one of the chief pathological features resulting from the PNI, causes further delay in regeneration by halting the synthesis of trophic factors and associated functional recovery [29–31]. The atrophy of denervated Gastrocnemius and Tibialis anterior muscle was reversed by *N. vomica*, with a statistically significant difference of **P < 0.01 (Tibialis anterior) ***P < 0.001 (Gastrocnemius). These results suggest that *N. vomica* seeds (crude form) retrieve the nerve functionality and help prevent muscular atrophy exacerbation.

The sciatic nerve is a mixed type of nerve that contains both sensory and motor nerve fibres. Therefore, measuring the sensory function regain is equally significant as of motor function following an injury. We observed an impact of *N. vomica* on retrieving the sensory functions as an observable alleviation in withdrawal latency of ipsilateral hind paw (^*###*^*P < 0.001*) was noted. Similarly, an early first lick response in *N. vomica* fed animal (^*#*^*P < 0.001*) also indicates the activation of pain fibres following the formalin injection. These findings highlight that *N. vomica* can be equally effective for the PNS, as previously reported for the CNS [13].

Oxidative stress contributes to the pathogenesis of a plethora of diseases and similar oxidative stress-based sequelae happen in the case of PNI [41]. It is one of the foremost hallmarks of neuronal damage causing the mitochondrial dysfunction, demyelination, neuroinflammation, and apoptosis. *N. vomica* enhances the anti-oxidative capacity by regulating the activity of anti-oxidative enzymes like superoxide dismutase (SOD) and catalase. There are many active constituents present in *N. vomica* which are accredited for their anti-oxidative capability [8, 42]. Our results are also consistent with these findings because we also observed a significant modulation of TAC (**P < 0.01) and TOS levels (*P < 0.05), and an increase in the activity of ARE and PON-1 enzymes. These enzymes are considered as anti-oxidants and can scavenge free radicals. Hyperglycaemia-induced oxidative stress is a well-recognized entity as a driving force for the development of numerous clinical problems. A balance between anti-oxidative processes and oxidative stress seems to be sensitive to glucose level as moderately elevated glucose level affects the TOS of the body [40]. Hyperglycaemia and impaired glycaemic control can initiate reductive stress which ultimately leads to oxidative stress that is a major mediator of local nerve injury causing delayed functional recovery [32–34]. Consequently, anti-oxidants help the body to ameliorate oxidative stress-induced cellular damages [43]. In our study, *N. vomica* supplementation improves glucose clearance in the treated group. We hypothesize that *N. vomica* somehow modulates the metabolic machinery at a cellular level for metabolizing glucose more efficiently that ultimately improves nerve regeneration. We also found that treatment with *N. vomica* affected blood cell counts, namely, platelets and granulocytes, which were found increased. Neutrophils, a subset of granulocytes, are critical for promoting axonal regeneration in the nervous system and are a major source of important factors in this regard [36, 37]. Accordingly, granulocyte colony-stimulating factor (G-CSF) prevents the early inflammation and subsequent development of neuropathic pain [44], and GM-CSF also improves the peripheral nerve regeneration following a sciatic nerve crush in a mouse model [45]. We noted a decrease in the circulating MID cells including monocytes in *N. vomica* treated group. This may depict margination of these cells or their migration to the site of injury to differentiate to resident macrophages and dendritic cells to modulate the recovery process [46]. Similarly, the platelet level was raised in the *N. vomica* group. Platelets are a rich source of growth factors (platelet-derived growth factor (PDGF), insulin-like growth factor I (IGF-1), fibroblast growth factor (FGF) and vascular endothelial growth factor (VEGF)) that may be associated with the accelerated repair of a nerve following an injury in the treated group. This statement can be strengthened by the positive association between platelet-rich plasma therapy and alleviated pathogenesis of PNI [47]. Collectively, present findings indicate a function recovery promoting effects of *N. vomica* against sciatic nerve injury and further work for the evaluation of its individual constituent(s) that may prove a valuable drug target for PNI and other myopathies.

## Conclusions

Current findings signify an impending role of *N. vomica* seeds powder to accelerate the neuronal function restoration following a mechanically induced nerve injury. These results indicate that *N. vomica* can be a good drug candidate for peripheral nerve regeneration in case of traumatic nerve injuries. Though this is a quite preliminary sort of study but it warrants further work to explore the effective potent constituents as well as underlying mechanisms that are responsible for this earlier functional rescue. The research platform is open for the studies, related to decoction from *N. vomica* seeds, for further exploration to develop cost-effective therapeutic products.

## Data Availability

The data sets used or analysed in this study are available from the corresponding author upon request. All data generated during this study are included in this manuscript.

## Abbreviations

PNI: Peripheral Nerve Injury
*N. vomica*: *Strychnos nux-vomica*
PD: Parkinson’s disease
AD: Alzheimer’s diseases
SFI: Sciatic Functional Index
HPL: Hot-plate latency
TOS: Total Oxidant Status
TAC: Total Antioxidant Capacity
TEAC: Trolox Equivalent Antioxidant Capacity
ABTS: 2,2′-azino-di-3-ethylbenzthiazoline sulfonate
PON-1: Paraoxonase activity
ARE: Arylesterase activity
GTT: Glucose Tolerance Test
G-CSF: Granulocyte colony-stimulating factor
